# Performance of the Trioplex real-time RT-PCR assay for detection of Zika, dengue, and chikungunya viruses

**DOI:** 10.1038/s41467-018-03772-1

**Published:** 2018-04-11

**Authors:** Gilberto A. Santiago, Jesús Vázquez, Sean Courtney, Katia Y. Matías, Lauren E. Andersen, Candimar Colón, Angela E. Butler, Rebecca Roulo, John Bowzard, Julie M. Villanueva, Jorge L. Muñoz-Jordan

**Affiliations:** 1 Division of Vector Borne Diseases, Dengue Branch, Centers for Disease Control and Prevention, National Centers for Emerging and Zoonotic Infectious Diseases, San Juan, PR 00920 USA; 2 Division of Preparedness and Emerging Infections, Centers for Disease Control and Prevention, National Centers for Emerging and Zoonotic Infectious Diseases, Atlanta, GA 30333 USA

## Abstract

The emergence and spread of Zika virus (ZIKV) presented a challenge to the diagnosis of ZIKV infections in areas with transmission of dengue (DENV) and chikungunya (CHIKV) viruses. To facilitate detection of ZIKV infections, and differentiate these infections from DENV and CHIKV, we developed the Trioplex real-time RT-PCR assay (Trioplex assay). Here, we describe the optimization of multiplex and singleplex formats of the assay for a variety of chemistries and instruments to facilitate global standardization and implementation. We evaluated the analytical performance of all Trioplex modalities for detection of these three pathogens in serum and whole blood, and for ZIKV in urine. The limit of detection for the three viruses and in different RNA-extraction modalities is near 10^3^ genome copy equivalents per milliliter (GCE/mL). Simultaneous testing of more than one specimen type from each patient provides a 6.4% additional diagnostic sensitivity. Overall, the high sensitivity of the Trioplex assay demonstrates the utility of this assay ascertaining Zika cases.

## Abstract (web summary)

The Trioplex real-time RT-PCR assay was developed for detection of Zika virus infections in areas with dengue and chikungunya transmission. Here, Santiago et al. describe the optimization and clinical performance of the assay, showing high sensitivity for detection and differentiation of the three viruses.

## Introduction

Since its arrival in the Americas in 2014, 48 countries and territories have reported Zika virus (ZIKV) transmission, affecting over 2 million humans, according to the Pan American Health Organization (PAHO)^1^. The virus is transmitted to humans primarily by the bite of infected *Aedes* spp. mosquitoes and recent reports have documented virus transmission through sexual contact^2–4^ and blood transfusions^5^. Generally, an overt clinical presentation is absent in most ZIKV infections, whereas symptomatic cases have similar clinical characteristics to DENV and CHIKV infections^6,7^. In infected pregnant women, symptomatic or not, ZIKV may infect the fetus and produce a range of developmental abnormalities such as microcephaly and other congenital and potentially fatal complications^8,9^. In addition, ZIKV infections have a strong association with Guillain–Barré syndrome^9–12^. Consequently, accurate detection of ZIKV is imperative for surveillance, disease management, and screening of pregnant women.

Public health laboratories, especially those located in endemic areas, have faced several challenges to the detection of ZIKV infection including the nonspecific clinical presentation which is similar to dengue and chikungunya disease, and the low viral loads detected during the acute phase of infection^5,13^. Furthermore, the identification of ZIKV infection is significantly hindered by the cross-reactivity exhibited between anti-ZIKV IgM and anti-DENV IgM antibodies, which makes the accurate determination of ZIKV infection by immunodiagnostic methods extremely difficult in regions with previous transmission of one or both pathogens^14–16^. Therefore, nucleic acid amplification tests (NAAT) have become a primary tool for the accurate diagnosis of ZIKV infections^17^. Before the introduction of ZIKV in the Americas, a limited number of laboratory-developed tests (LDTs) were described^18–22^ but had not been extensively evaluated for the variety of human clinical specimens where ZIKV RNA is known to be present^23–26^. Given varied testing capacities and regulations, many public health laboratories required a standardized test with extensive performance evaluation to facilitate validation and implementation.

In response to the diagnostic challenges presented by the ZIKV epidemic, the Centers for Disease Control and Prevention (CDC) developed the Trioplex real-time reverse transcriptase-polymerase chain reaction (real-time RT-PCR) assay (Trioplex assay) for the concurrent detection of ZIKV, DENV, and CHIKV RNA in human serum, whole blood in ethylenediaminetetraacetic acid (EDTA) and cerebrospinal fluid (CSF) or the sole detection of ZIKV in human urine or amniotic fluid^27^. The performance of the Trioplex assay including a selection of RNA extraction methods, PCR chemistries, and real-time PCR instruments was evaluated to facilitate implementation and standardization across public health laboratories globally^27^. In March of 2016, the assay received Emergency Use Authorization (EUA) by the United States Food and Drug Administration (FDA)^28^.

Here, we present the analytical and clinical performance evaluations of the Trioplex assay, and document its limit of detection (LoD) and utility as a flexible test in available diagnostic devices, allowing most public health laboratories to detect Zika, dengue, and chikungunya cases globally

## Results

### Multiplex and singleplex detection of target virus RNA

**Fig. 1:**
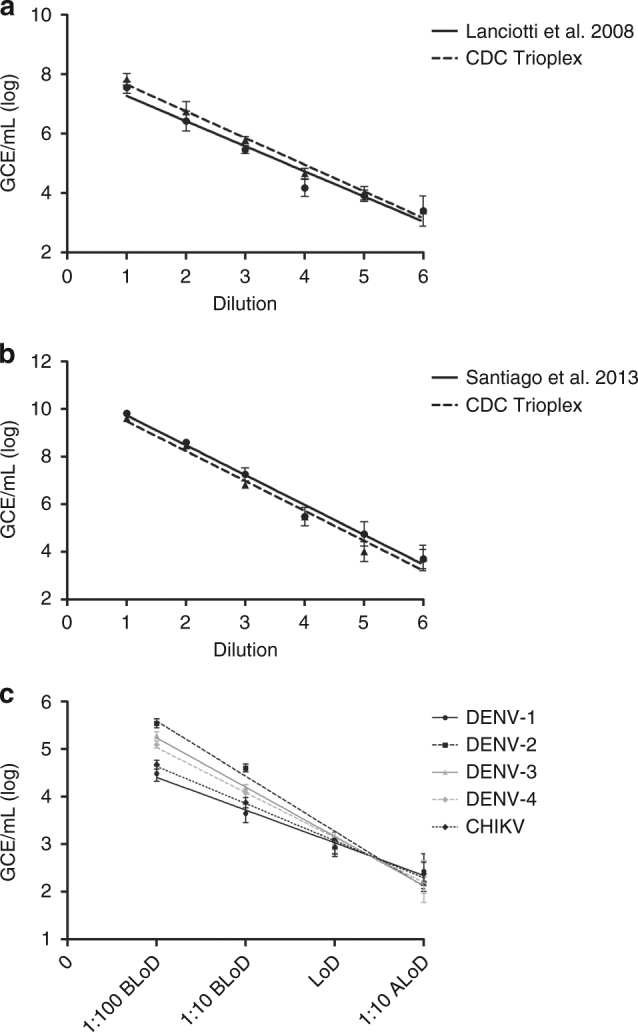
Analytical performance of the Trioplex assay in multiplex format for all target viruses. Normal human serum was spiked with Zika virus, dengue virus type 1 (DENV-1), dengue virus type 2 (DENV-2), dengue virus type 3 (DENV-3) or dengue virus type 4 (DENV-4). Six serial dilutions (1:10) were tested to compare ZIKV detection performance of the Trioplex assay with the CDC ZIKV reference assay^18^ (**a**) or DENV detection with the CDC DENV reference assay^29^ (**b**). Data for DENV-4 is shown. Straight line represents linear regression of CDC reference test. Dashed line represents linear regression for Trioplex assay. Trioplex assay limit of detection evaluated for each DENV serotype and chikungunya virus testing 20 replicates per dilutions: 1:100 before LoD (BLoD), 1:10 before LoD (BLoD), LoD and 1:10 after LoD (ALoD) (**c**). Mean genome copy equivalents per milliliter (GCE/mL) of viral RNA are displayed at each dilution. Error bars represent GCE/mL standard deviation

The Trioplex assay was designed for the detection of ZIKV, DENV, and CHIKV RNA in human diagnostic specimens. Each virus-specific set of oligonucleotides was optimized individually, and sets were combined into a one-step, single-reaction assay, and compared with other CDC reference assays^18,29^. To evaluate the performance and determine the LoD of the Trioplex assay compared to other related CDC assays, stocks of live, infectious ZIKV, DENV, and CHIKV previously obtained in tissue culture at a titer of 10^6^ plaque forming units per milliliter (pfu/mL)^30^ were suspended in normal human serum and quantified with standardized virus-specific RNA transcripts. Some of these analyses and comparative studies are presented along with more characteristics and operational details of the Trioplex in publicly available FDA resources^28^. The LoD was defined as the lowest dilution at which the assay detected ≥95% of contrived replicates. For an initial evaluation of the Trioplex assay, virus suspensions were diluted in normal human serum in 1:10 series six times and all RNA extractions were performed using the small volume (0.2 mL) methods on the MagNA Pure LC 2.0 (Roche) instrument (Fig. 1).

The Trioplex assay (in multiplex format) was compared with a previously published CDC ZIKV real-time RT-PCR reference assay^18^ (Fig. 1a). Results showed that the Trioplex assay and the CDC ZIKV reference assay perform similarly across the dilution series and have comparable limits of detection. The performance of the Trioplex assay in detecting of DENV was next compared to a published CDC DENV-1-4 real-time RT-PCR reference assay^29^ (Fig. 1b). The Trioplex assay includes a novel oligonucleotide set that detects all DENV serotypes without individual serotype identification. Quantified stocks of each DENV serotype were suspended in normal human serum, diluted in 1:10 series six times and each DENV-1-4 replicates were evaluated by individual serotype. Results showed that the Trioplex assay performs similarly to the CDC DENV reference assay across the dilution series of each DENV serotype (comparison of DENV-4 dilutions are shown in Fig. 1b). To further assess the LoD of the Trioplex assay for DENV and CHIKV, quantified stocks of target virus suspended in normal human serum were diluted to a range of four 10-fold dilutions before, and after the LoD and 20 replicates per dilution per target virus were tested (Fig. 1c). These comparisons showed that the LoD of the Trioplex assay for DENV-1-4 ranges from 4 × 10^3^ to 1 × 10^4^ GCE/mL and is comparable to other CDC DENV reference assays^29,31^. Similarly, the LoD of CHIKV was determined to range between 5 × 10^3^ to 4 × 10^4^ GCE/mL.

### Evaluation of RNA extraction and PCR instrumentation

Serum and urine are the most frequently collected clinical specimens for Zika virus RNA detection since the emergence of the epidemic and continue to be the most frequently tested^24,26^. With the objective to include a variety of RNA extraction methods and real-time PCR instruments for the Trioplex assay, the analytical performance of the QIAamp Viral RNA Mini kit (Qiagen), a manual extraction method that utilizes silica-based filter columns, was compared to the automated high-throughput magnetic bead extraction systems, including the MagNA Pure LC 2.0 (Roche), the MagNA Pure 96 (Roche), MagNA Pure Compact (Roche), and NucliSENS® easyMag® (bioMérieux). Two real-time PCR instruments were also evaluated: the ABI 7500 Fast Dx (ThermoFisher) and the QuantStudio Dx (ThemoFisher). To evaluate the performance characteristics and standardize the use of the Trioplex assay paired with each system; the LoD of the assay under each system combination was evaluated.

Similar to the previous evaluations, analytical panels of normal human serum or urine pooled from healthy pre-tested human donors were suspended with quantified stocks of ZIKV. Viral suspensions were diluted in series 1:10, and each dilution series was tested in triplicate in an initial range-finding study to identify the LoD. All RNA extractions were performed using the small volume (0.2 mL) methods of each extraction system and the LoD dilution was confirmed by testing 20 replicates per dilution surrounding the tentative LoD.

**Fig. 2:**
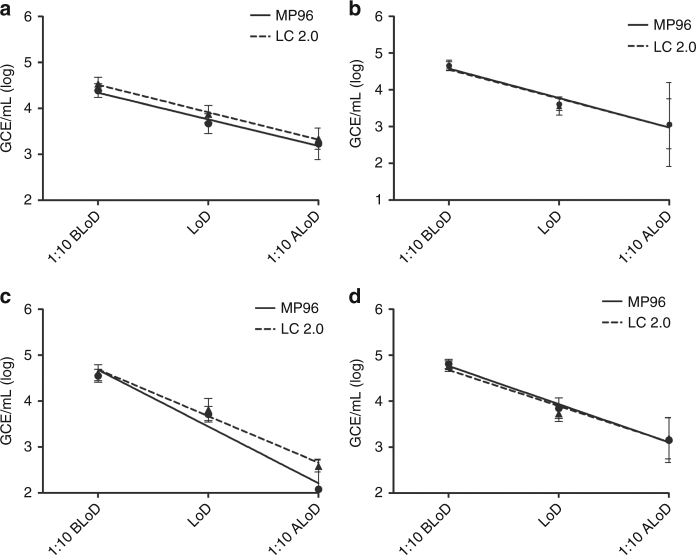
Analytical performance comparison between MP96 and LC 2.0 RNA extraction platforms in serum and urine. **a** Normal human serum or **b** urine pooled from healthy donors was contrived with ZIKV at a dilution of 1:10 before the limit of detection (1:10 BLoD), at the limit of detection (LoD), and at 1:10 after the limit of detection (1:10 ALoD). Twenty replicates of each dilution were extracted using the small volume protocol (0.2 mL) and were tested with the Trioplex assay on the ABI7500 Fast Dx instrument. The same serum (**c**) and urine (**d**) dilutions were tested on the QuantStudio Dx instrument. Mean genome copy equivalents per milliliter (GCE/mL) of viral RNA detected are displayed at each dilution. Error bars represent GCE/mL standard deviation. Straight line represents linear regression of MP96 platform (Roche). Dashed line represents linear regression of LC 2.0 platform (Roche)

In Fig. 2, ZIKV RNA was extracted from serum (Fig. 2a, c) or urine (Fig. 2b, d) using both the MagNA Pure LC 2.0 and MagNA Pure 96, and evaluated using the ABI 7500 Fast Dx (Fig. 2a, b) or the QuantStudio Dx (Fig. 2c, d) real-time PCR platform. These results indicate that the performance of both automated RNA extraction systems with spiked serum and urine specimens is comparable between both specimen types and on both PCR platforms. The LoD for ZIKV RNA using the ABI 7500 Fast Dx with serum specimens extracted from both systems ranged from 5 × 10^3^ to 1 × 10^4^ GCE/mL (Fig. 2a) and from 3 × 10^3^ to 4 × 10^3^ GCE/mL in urine (Fig. 2b). Similarly, the LoD for ZIKV RNA using the QuantStudio Dx with serum specimens extracted from both systems ranged from 5 × 10^3^ to 6 × 10^3^ GCE/mL (Fig. 2c) and from 5 × 10^3^ to 7 × 10^3^ GCE/mL in urine (Fig. 2d). Compared to previously reported data^28^, the performance of the MagNA Pure LC 2.0 and MagNA Pure 96 was comparable to that observed on the MagNA Pure Compact and NucliSENS® easyMag® instruments^27^. The manual extraction method produced similar results but, with a slightly higher LoD than the automated systems^27^. However, due to the low throughput of this method and complexity of the comparative analysis, this manual method was not evaluated further.

The performance of an alternative RT-PCR master mix, qScript™ One-Step qRT-PCR kit, Low Rox™ (Quanta) real-time RT-PCR master mix, was evaluated on the ABI 7500 Fast Dx and QuantStudio Dx instruments using the same RNA that was tested in the previous study. Performance and the LoD observed were equivalent to the data obtained with SuperScript® III Platinum® One-Step qRT-PCR System without ROX (ThermoFisher)^27^.

### Evaluation of RNA extraction and qPCR instruments for whole blood

**Fig. 3:**
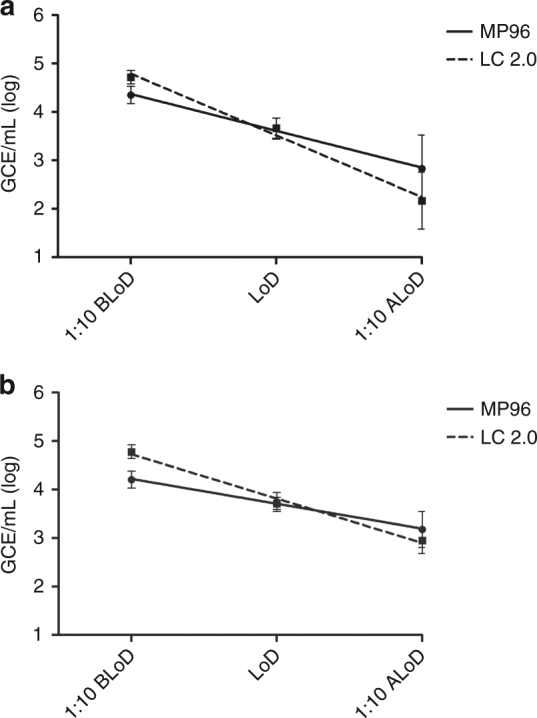
Analytical performance comparison between MP96 and LC 2.0 RNA extraction platforms in whole blood (EDTA). A pool of whole blood donated by healthy donors was contrived with ZIKV at a dilution of 1:10 before the limit of detection (1:10 BLoD), at the limit of detection (LoD) and at 1:10 after the limit of detection (1:10 ALoD). Twenty replicas of every dilution were extracted using small volume protocol (0.2 mL) and tested with Trioplex assay on the ABI7500 Fast Dx instrument (**a**) or in the QuantStudio Dx instrument (**b**). Mean genome copy equivalents per milliliter (GCE/mL) of viral RNA detected are displayed at each dilution. Error bars represent GCE/mL standard deviation. Straight line represents linear regression of MP96 platform (Roche). Dashed line represents linear regression of LC 2.0 platform (Roche)

ZIKV RNA detection in whole blood has been recently documented^32^, however, no ZIKV molecular diagnostic assays have been adapted to test this specimen type. Whole blood-EDTA presents a challenge for NAATs considering the viscosity, potential presence of PCR inhibitors and high cellular content. In order to extract ZIKV RNA using automated RNA extraction systems, we developed an external lysis protocol performed prior to extraction that facilitates the neutralization of potential PCR inhibitors and prevents the clogging of the automated liquid transfer systems. Performance of the Trioplex assay was evaluated using RNA extractions from the MagNA Pure LC 2.0, MagNA Pure 96, MagNA Pure Compact, and NucliSENS® easyMag® platforms and run on the ABI 7500 Fast Dx and QuantStudio Dx real-time PCR instruments. Whole blood from healthy, pre-tested human donors was pooled and spiked with quantified stocks of ZIKV. Similar to previous evaluations, virus suspensions were diluted in series 1:10 in an initial range-finding study to identify a tentative LoD. The LoD was then confirmed by testing 20 replicates at the selected dilutions surrounding the tentative LoD. Prior to testing, samples were treated with the external lysis buffer and extracted RNA using the small volume (0.2 mL) protocols of the automated extraction methods. Here, RNA was extracted using the MagNA Pure LC 2.0 and the MagNA Pure 96 instruments and analyzed on the ABI 7500 Fast Dx (Fig. 3a) and from the QuantStudio Dx (Fig. 3b). These data between all automated extraction systems and between real-time PCR instruments determined the LoDs to be comparable, ranging from 6 × 10^3^ to 9 × 10^3^ GCE/mL with ZIKV RNA. Furthermore, a previous study evaluating DENV and CHIKV suspended in whole blood-EDTA found a comparable LoD for each target virus^27^ as was observed here using spiked serum and urine.

In order to further evaluate testing of whole blood-EDTA and determine the stability of ZIKV RNA in this specimen type over time after collection, whole blood and serum samples were collected from 34 patients that had ZIKV-positive sera using the Trioplex assay. An aliquot of each fresh whole blood sample was immediately frozen at −20 °C and two other aliquots were stored at 4 °C. The refrigerated aliquots were tested at days 3 and 9 post-storage, respectively, and the frozen aliquot was tested at day 10. Ninety-seven percent (97%, 33/34) of the samples tested positive with a <1 log change in copy number under any of the tested storage conditions, thus suggesting that the viral RNA remains detectable up to 9 days at 4 °C and at least 10 days frozen at −20 °C (Supplementary Table 1).

### Small and large volume RNA extraction validation

**Fig. 4:**
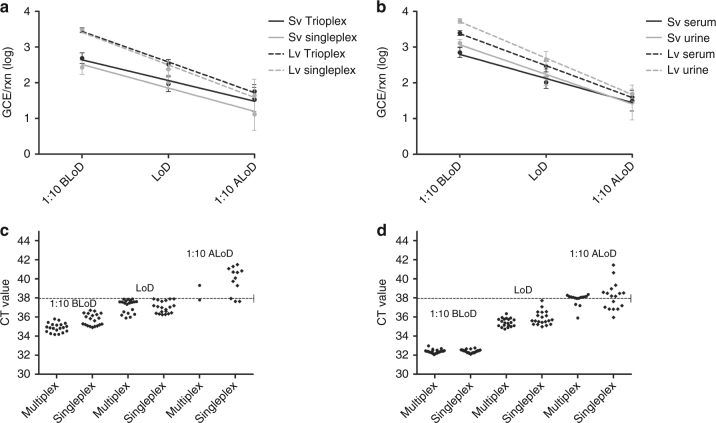
Analytical performance comparison between Trioplex assay multiplex and the ZIKV singleplex format assay using small volume and large volume RNA extraction. Normal human serum or urine was contrived with ZIKV at a dilution of 1:10 before the limit of detection (1:10 BLoD), at the limit of detection (LoD), and at 1:10 after the limit of detection (1:10 ALoD). Twenty replicates of every dilution were extracted using the MagNA Pure 96 instrument (Roche) and tested by Trioplex assay multiplex or ZIKV singleplex format assay on the ABI 7500 Fast Dx or the QuantStudio Dx instruments. **a** Compares the mean genome copy equivalents per PCR reaction (GCE/rxn) of viral RNA extracted from serum at each dilution on the ABI 7500 Fast Dx instrument. A linear regression was plotted for multiplex with small volume protocol (Sv) (0.2 mL) (black straight line), singleplex assay with small volume protocol (gray straight line), multiplex with large volume protocol (Lv) (1 mL) (black dashed line). and singleplex assay with large volume protocol (gray dashed line). **b** Compares the mean genome copy equivalents per PCR reaction (GCE/rxn) of viral RNA extracted from serum or urine at each dilution on the QuantStudio Dx instrument. A linear regression was plotted for multiplex with small volume protocol serum (Sv) (black straight line), multiplex with large volume protocol serum (Lv) (gray straight line), multiplex with small volume protocol urine (Lv) (black dashed line), and multiplex with large volume protocol urine (gray dashed line). Error bars represent GCE/mL standard deviation. The CT values for every dilution replicate in serum tested was plotted for **c** small volume and **d** large volume extractions

Detecting ZIKV is critical in diagnosing pregnant women who may or may not have symptoms associated with ZIKV infections. With the objective to increase the sensitivity of the Trioplex assay, the sample input volume was next assessed in an effort to increase the concentration of target RNA in the eluate by increasing the volume of sample tested. To test this hypothesis, RNA was extracted from normal human serum suspended with ZIKV, generating a panel to evaluate the LoD as described in the previous sections. RNA was extracted using the MagNA Pure 96 using the small (0.2 mL) or large (1 mL) volume methods and assessed on the ABI 7500 Fast Dx in both the multiplex and singleplex formats (Fig. 4a). To facilitate visualization and to normalize RNA quantification between validations, data are expressed in GCE detected per PCR reaction (GCE/rxn) regardless of extraction volume method. An approximate increase of 0.5 log GCE/rxn was obtained when using large volume RNA extractions in normal human serum as compared to small volume extractions (Fig. 4a). The LoD between multiplex and singleplex formats was comparable as shown in the preceding Trioplex assay evaluations. This evaluation was extended to include a comparison between contrived serum and urine specimens tested in the QuantStudio Dx instrument in multiplex format. Results from this evaluation confirmed the LoD previously obtained in the MagNA Pure 96 small volume extraction for serum and urine; however, an approximate increase of 0.7 log GCE/rxn was obtained in urine when using large volume RNA extractions (Fig. 4b). The benefit of large volume RNA extractions on increasing diagnostic sensitivities is even more evident when the CT value of each individual PCR reaction is compared between extraction methods. The CT values of each individual multiplex or singleplex PCR reaction in the LoD dilution range obtained from small volume (Fig. 4c) or large volume extractions (Fig. 4d) were plotted. Figure 4c,d shows the general decrease in CT values that corresponds to an increase in copy number. At the LoD dilution, the median CTs for small volume extraction were 37.52, 95% CI 36.85, 37.49 (multiplex format) and 37.02, 95% CI 36.74, 37.31 (singleplex format) (Fig. 4c), whereas the median CTs for large volume extraction were 35.09, 95% CI 35.24, 35.65 (multiplex format) and 35.28, 95% CI 35.54, 36.21 (singleplex format) (Fig. 4d). Large volume extractions (Fig. 4d) allowed detection of more replicates in the dilution after LoD including more replicates with CT ≤ 38 or detectable amplification curves with CT between 38 and 42. To document these changes of sensitivity, all CT values obtained have been included, though only CTs < 38 are considered positive for diagnostic purposes.

### Specific detection of target virus RNA

The specificity target detection is critical for every diagnostic assay, especially when the assay is intended to accurately discriminate between diseases caused by similar pathogens or similar clinical presentations. To evaluate the specificity of the Trioplex real-time RT-PCR assay, a panel was generated that contained different combinations of ZIKV, DENV or CHIKV suspended in a pool of human serum samples obtained from patients with febrile illness but had previously tested negative by PCR for any of the virus targets tested in this study. Multiple combinations of each target virus were mixed at concentrations ranging from 10^5^ to 10^8^ GCE/mL, RNA was extracted using the MagNA Pure LC 2.0 small volume protocol (0.2 mL) from 5–10 replicates per mix and tested with the Trioplex assay in multiplex format in the ABI 7500 Fast Dx instrument (Table 1). Fifty replicates of mock-suspended serum samples were also tested to determine the rate of false-positive detection. Results showed that each intended target virus and virus combination was identified correctly at the expected concentration and that all 50 mock serum replicates tested negative (Table 1)^27^. Overall, these data demonstrate that the presence of multiple virus targets does not affect target detection in this assay.

**Table 1:** Mixed virus RNA detection in serum from acute febrile illness patients negative for dengue, chikungunya, and Zika viruses

		Mean GCE/mL		
Target mix	# replicates	DENV	CHIKV	ZIKV
ZIKV	5	Negative	Negative	2.71E + 06
DENV	5	8.91E + 05	Negative	Negative
CHIKV	5	Negative	3.67E + 06	Negative
ZIKV	5	Negative	Negative	4.70E + 07
DENV	5	1.34E + 08	Negative	Negative
CHIKV	5	Negative	4.00E + 07	Negative
DENV + ZIKV	10	1.60E + 08	Negative	4.03E + 06
CHIKV + ZIKV	10	Negative	4.66E + 07	4.24E + 06
DENV + ZIKV	10	7.81E + 05	Negative	5.78E + 07
DENV + CHIKV	10	1.43E + 06	4.16E + 07	Negative
CHIKV + ZIKV	10	Negative	7.16E + 06	5.90E + 07
DENV + CHIKV	10	1.81E + 08	3.90E + 06	Negative
DENV + CHIKV + ZIKV	10	2.16E + 08	5.80E + 07	6.01E + 07
Mock	50	Negative	Negative	Negative

To further evaluate cross-reactivity of the assay with other non-target, genetically similar viruses, RNA was extracted from laboratory stocks of West Nile virus, Yellow Fever virus, and St. Louis encephalitis virus and three 10-fold dilutions of each were tested in duplicate near the LoD with the Trioplex assay in multiplex format (Supplementary Table 2). Results from this study showed that only specific target virus controls were amplified and no non-specific signals were detected with any of the non-target virus RNA.

### Clinical performance of the Trioplex assay on samples from symptomatic patients

In order to evaluate the sensitivity of the Trioplex assay detecting ZIKV RNA in clinical specimens, we randomly selected 155 clinical samples from CDC’s surveillance system in Puerto Rico, which detects acute febrile cases (all ages) with and without severe signs or symptoms across the island that were previously collected during epidemic periods of dengue, chikungunya, and Zika transmission between 2012 and 2016. Specimens included 82 sera positive for DENV (24 DENV-1, 22 DENV-2, 25 DENV-3, and 11 DENV-4), 13 from CHIKV cases, 20 from ZIKV cases, and 40 negative cases specimens (no virus or antibodies identified with in-house CDC reference tests). RNA was extracted with the MagNA Pure 96 small volume protocol and tested with the Trioplex assay in multiplex format on the ABI 7500 Fast Dx instrument (Table 2). A 95% positive agreement with previous diagnostic determination was observed for ZIKV (19/20), 100% CHIKV (13/13) specimens, and 100% (82/82) for DENV specimens. Similarly, a 99.2% (134/135) negative agreement was observed on ZIKV testing, 100% (73/73) on DENV testing, and 100% (142/142) on CHIKV testing.

**Table 2:** Performance of Trioplex with archived clinical serum specimens

Specimen category	Tested	ZIKV^a^ positive	DENV^a^ positive	CHIKV^a^ positive	Positive percent agreement	Negative percent agreement
Zika	20	19/20^b^	0/20	0/20	95% (19/20) (76.4–99.1%)	99.2% (134/135) (95.9–99.9%)
Dengue	82	0/82	82/82	0/82	100% (82/82) (95.5–100%)	100% (73/73) (95–100%)
Chikungunya	13	0/13	0/13	13/13	100% (13/13) (77.2–100%)	100% (142/142) (97.4–100%)
Negative	40	1/40^b^	0/40	0/40	N/A	N/A

N/A not applicable

^a^ Trioplex component result

^b^ One Zika specimen tested negative with the Trioplex assay but tested positive (CT 34.4) with the CDC Zika NS3 assay^18^. This specimen also tested positive for Zika IgM with the CDC Zika IgM Mac ELIZA

**Fig. 5:**
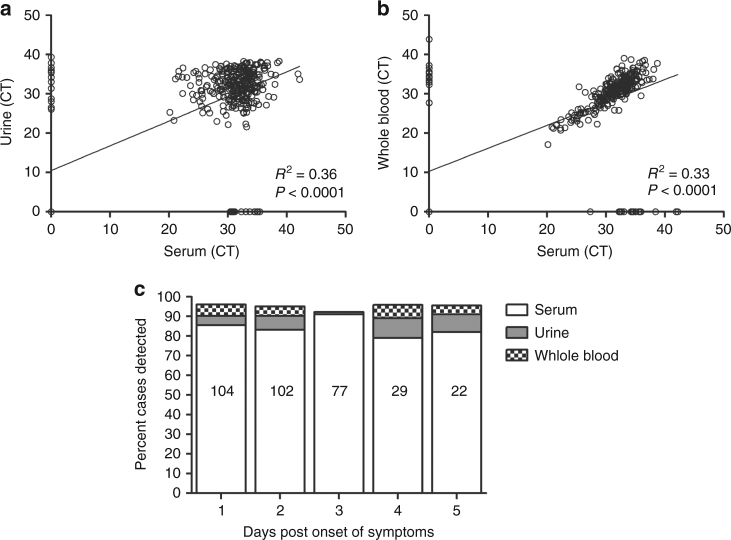
Clinical performance of the Trioplex assay across specimen types. Clinical specimens collected concurrently from 373 cases with previous Zika determination in the acute stage were tested. RNA was extracted with the MagNA Pure 96 small volume external lysis protocol from 373 case-paired serum, 373 urine, and 345 whole blood-EDTA specimens and tested with the Trioplex assay in multiplex format in the ABI 7500 Fast Dx instrument. **a** Correlation of CT values between case-matching serum and urine specimens; *R*^2^ = 0.36 *p* < 0.0001. **b** Correlation of CT values between case-matching serum and whole blood-EDTA specimens; *R*^2^ = 0.33 *p* < 0.0001. **c** Dataset separated by DPO 1–5 and percent cases detected by the Trioplex assay in every specimen type. The white bar represents the percent of cases with ZIKV detected in serum specimens. The gray bar represents the percent of ZIKV-positive urine specimens that resulted ZIKV-negative in the matching serum specimen, and the checkered box represents the percent of ZIKV-positive whole blood specimens resulted ZIKV-negative in the matching serum specimen. Both gray and checkered boxes represent the added value of testing more than one specimen type with the Trioplex assay

To further evaluate ZIKV RNA detection across clinical specimen types, we prospectively selected 373 ZIKV cases with a Trioplex positive result identified in Puerto Rico during the 2016 epidemic in at least one of three concurrently collected serum, urine, and whole blood-EDTA sample during the first 6 days following onset of symptoms. These samples were obtained from patients from CDC’s routine surveillance system with signs and symptoms of Zika illness with or without fever. In addition, each case tested positive for anti-ZIKV IgM in the convalescent phase using the CDC Zika MAC-ELIZA. The RNA was extracted from these samples using the MagNA Pure 96 small volume external lysis protocol. The Trioplex assay detected ZIKV RNA in 85% (317/373) of the serum specimens, 83% (311/373) of the urine specimens, and 82% (285/347) of the whole blood-EDTA specimens (Fig. 5). In addition, every specimen was also tested with the RP internal control reaction and invalid specimens were discarded from the study. Descriptive statistics for each specimen type including the internal control RP reaction were calculated (Supplementary Table 3). A positive and significant correlation was observed between the Trioplex CT values obtained between the serum and urine specimens (Fig. 5a), and between serum and whole blood-EDTA specimens (Fig. 5b). Furthermore, by separating the case dataset by days post-onset of symptoms (DPO), the data showed that testing more than one clinical specimen type per case provides added diagnostic value by increasing sensitivity up to an average of 6.4% during the first 5 days of symptom onset (Fig. 5c).

## Discussion

Amidst the emergence of the 2016 ZIKV epidemic in the Americas, the CDC developed the Trioplex real-time RT-PCR assay to meet the demands of public health laboratories for a standardized, sensitive, and accurate molecular diagnostic test. Following the approval of the EUA issued by the FDA in March 2016, the Trioplex assay has been deployed by the CDC to over 200 public health laboratories in the USA and internationally. This assay was designed to detect ZIKV RNA, as well as DENV and CHIKV viruses, which frequently co-circulate in tropical and sub-tropical regions; thus presenting a useful tool for accurate discrimination between co-endemic diseases of similar clinical manifestation. To seek authorization for emergency use, this assay was evaluated in serum, blood, and urine. The test is also authorized for use in CSF and amniotic fluid by reliance in our serum evaluation. To facilitate large-scale implementation for the Zika epidemic of 2016–17, the test was developed to function in multiple molecular diagnostics systems frequently found in public health laboratories. More than 300 public health laboratories worldwide have run nearly 2 million Trioplex tests to assist during this emergency.

The performance of the assay was standardized across a combination of methods and clinical specimen types to guarantee reproducibility. Performance characteristics of the Trioplex assay were verified using four automated high-throughput and one manual RNA extraction methods, two one-step real-time RT-PCR master mixes and two real-time PCR thermocyclers with serum, urine and whole blood-EDTA specimen types. The LoD of the assay was found to be comparable between the combination of methods, between specimen types, and between target viruses ranging from 3 × 10^3^ to 4 × 10^4^ GCE/mL. Data presented here showed that large volume (1 mL) RNA extraction methods provide the highest sensitivity format; thereby increasing assay sensitivity by 0.5 logs and 0.7 logs GCE/mL in serum and urine, respectively. Furthermore, the validations presented in this study demonstrate that the performance and LoD of the multiplex and singleplex formats is equivalent for each target virus for this assay. Independent studies have confirmed that the sensitivity of the Trioplex assay in its high input modality is the most sensitive modality of this test and comparable other CDC and non-CDC tests^33–36^. Because virus concentrations during ZIKV infections are low, increasing assay sensitivity is of paramount importance^33–36^. Generally, increasing sample RNA input provides higher sensitivity; however, our method is limited to the use of conventional real-time RT-PCR systems that utilize commercially available ancillary reagents at fixed concentrations. We continue to evaluate new systems and technologies to increase assay sensitivity further. The Trioplex has been compared with a number of blood donation screening systems, which are more sensitive due to the use of large-volume blood sample and a higher proportion of the RNA extract^33^. For this reason, and because the Trioplex assay has been evaluated only in symptomatic individuals, the Trioplex assay is not intended for blood donation screening.

The clinical evaluation presented in this study demonstrates that the Trioplex assay functions as expected in the clinical setting, detecting viral RNA in ~85% of the serum specimens from symptomatic patients with previous immunodiagnostic determination. In contrast, the Trioplex assay sensitivity (percent positives) observed in urine specimens is moderately inferior to the clinical sensitivity of Zika RNA detection reported by other public health laboratories^24,37^. The foundation of this dissimilarity remains uncertain; however, urine specimen collection, handling, and processing prior to diagnostic testing could influence assay sensitivity considering environmental temperatures, low pH, or presence of potential PCR inhibitors^38^. As for every NAAT, performance, sensitivity, and reproducibility are limited by specimen quality and interpretation of results. Each target virus generates real-time RT-PCR amplification patterns that are characteristic of the virus that must be considered when analyzing data to avoid reporting of false-positive results.

In conclusion, this evaluation demonstrates the capacity of the Trioplex real-time RT-PCR assay to detect dengue, chikungunya, and Zika virus RNA with high sensitivity in a variety of clinical specimens. The FDA also approved the use of the Trioplex assay for ZIKV detection in amniotic fluid and CSF based on the cell-free nature of these diagnostic specimens. The utility of this assay to function as a molecular diagnostic test and as research tool has been documented throughout the Zika epidemic in the Americas^26,39,40^. During the 2016–2017 epidemic in Puerto Rico, the Trioplex assay detected over 30,000 Zika infections, accounting for ~85% of the Zika sero-positive^41^. The validation of the assay across an array of methods and formats provides the user with highly versatile but standardized operating platforms readily adaptable to clinical and reference diagnostic laboratories. Though the Trioplex assay was developed to serve as a discriminatory diagnostic tool for regions where DENV, CHIKV, and ZIKV transmission has been documented, this assay also functions as a screening tool for travelers returning from such regions. Considering the challenges currently faced with serological assays for Zika detection—low sensitivity and specificity, the Trioplex assay presents a promising test that can be incorporated into diagnostic and surveillance algorithms for high-risk populations such as asymptomatic pregnant women or the general symptomatic population.

## Methods

### Diagnostic specimens and ethics statement

Clinical specimens were obtained through the Sentinel Enhanced Dengue Surveillance System (SEDDS); a prospective surveillance of acute febrile illness among patients presenting to the study clinic in Ponce, PR. Participating patients provided written consent for collection and testing of serum, urine, and whole blood in EDTA during the acute symptomatic period under the guidelines approved by the CDC and Ponce School of Medicine institutional review boards (IRB) protocol 6848. Specimens were de-linked from patient identifiers according to IRB protocol.

### Virus stock quantification

In vitro transcribed RNA was used as the copy number control to quantify stocks of each virus evaluated in the analytical panels as previously described^29^. Briefly, DNA templates were generated from each dengue virus serotype, DENV-1 Puerto Rico strain 1998, DENV-2 Puerto Rico strain 1998, DENV-3 Puerto Rico strain 2004, DENV-4 Puerto Rico strain 1998, CHIKV Puerto Rico strain 2014, and ZIKV French Polynesia strain 2013 obtained from the CDC Dengue Branch archival reference collection, amplified with the Trioplex real-time RT-PCR assay, and cloned into the pCR-II TOPO TA vector (ThermoFisher). Target RNA was transcribed with T7 RNA polymerase using AmpliScribe T7 Flash transcription kit (Lucigen Corp.). The resulting RNA was quantified with a spectrophotometer and the remaining template DNA was digested using the TURBO DNA-free kit (ThermoFisher). Standard curves were generated and tested with the Trioplex real-time RT-PCR assay on each qPCR instrument, ABI 7500 Fast Dx (ThermoFisher), and QuantStudio Dx (ThermoFisher). A linear regression was calculated assuming that every PCR amplicon is equivalent to a complete virus genome and the copy number is directly expressed as GCE per PCR reaction (GCE/rxn). To determine GCE per milliliter (GCE/mL) of sample, the RNA extraction method including sample volume input, RNA elution volume, and RNA input into PCR reaction were considered into the conversion.

### RNA extraction methods

This study selected to evaluate both manual and automated viral RNA extraction systems commonly available in public health laboratories. For manual viral RNA extraction from cell-free specimens (serum and urine), the silica-based filter column systems QIAamp Viral RNA Mini kit or QIAamp DSP Viral RNA Mini kit (Qiagen) was selected. The manufacturer suggested spin protocol was followed, where 140 µL of sample volume are extracted and RNA is eluted into 60 µL of AVE buffer. The automated systems evaluated in this study utilize high-throughput magnetic beads nucleic acid extraction methods and allow extraction from small and large volumes of diagnostic sample. For viral RNA extraction from serum and urine on the MagNA Pure LC 2.0 automated system (Roche Diagnostics), the MagNA Pure LC Total Nucleic Acid isolation kit-small volume was used and the manufacturer Total_NA_Variable_Elution protocol with 200 µL sample input volume and RNA elution into 60 µL of elution buffer. This protocol performs sample lysis internally within the enclosed system platform. To extract viral RNA using external lysis, 200 µL of sample volume was mixed with 300 µL of MagNA Pure LC lysis buffer inside a biological safety cabinet and then loaded into the system selecting the Total_NA_External_Lysis protocol using a 500 µL of sample input volume and RNA elution into 60 µL of elution buffer. To perform large volume extractions on the MagNA Pure LC 2.0 automated system, the MagNA Pure LC Total Nucleic Acid isolation kit-large volume was used and the manufacturer Total NA Variable Elution protocol was followed using a 1000 µL sample input volume and RNA elution into 100 µL of elution buffer. For viral RNA extraction from serum and urine on the MagNa Pure 96 automated system (Roche Diagnostics), the MagNA Pure 96 DNA and Viral NA Small volume kit was used and followed the manufacturer Viral_NA_Universal_SV_3.0 or 3.1 internal lysis protocol under the DNA/Viral_NA_SV_2.0 program was followed using a 200 µL sample input volume and RNA elution into 100 µL of elution buffer. To extract viral RNA using external lysis, 200 µL of sample was mixed with 250 µL of MagNA Pure 96 lysis buffer inside a biological safety cabinet and then loaded into the system selecting the Viral_NA_plasma_ext_lys_SV_3.0 or 3.1 protocol using a 450 µL sample input volume and RNA elution into 100 µL of elution buffer. To perform large volume extractions on the MagNA Pure 96 automated system, the MagNA Pure 96 DNA and Viral NA large volume kit was used and the manufacturer Viral_NA_Universal_LV_1000_3.0.1 or 3.1 internal lysis protocol under the DNA/Viral_NA_LV_2.0 program was followed using a 1000 µL sample input volume and RNA elution into 100 µL of elution buffer. The external lysis option is only available on the MagNA Pure 96 large volume protocol for a 500 µL sample volume input, not evaluated in this study. For viral RNA extraction from serum and urine on the MagNA Pure Compact automated system (Roche Diagnostics), the MagNA Pure Compact Nucleic Acid Isolation Kit I was used and the manufacturer Total_NA_Plasma_external_lysis_V3_2 protocol was followed using a 200 µL sample input volume and RNA elution into 100 µL of elution buffer. To perform large volume extractions on the MagNA Pure Compact, the the MagNA Pure Compact Nucleic Acid Isolation Kit I-large volume was used and the manufacturer Total_NA_Plasma_1000 protocol was followed using a 1000 µL sample input volume and RNA elution into 100 µL of elution buffer. The NucliSENS® easyMag® (bioMérieux) differs from the Roche systems in that it does not use kits but instead individual reagents. The reagents used for this study include Buffer 1–3, lysis buffer, and magnetic silica as recommended by the manufacturer. For small volume extractions of viral RNA from serum and urine, the Generic 2.0.1 protocol was selected using a 200 µL sample volume input and RNA elution into 100 µL of elution buffer. Similarly, for large volume extractions, the Generic 2.0.1 protocol was selected using a 1000 µL sample volume input and RNA elution into 100 µL of elution buffer. Due to the viscosity and high human cellular and genomic content of whole blood-EDTA specimens, 200 µL of sample was mixed with 300 or 250 µL of external lysis buffer (depending on extraction instrument) and incubated at room temperature for 15–20 minutes prior to loading into any of the MagNA Pure systems. Viral RNA extractions from whole blood-EDTA specimens were not evaluated using the NucliSENS® easyMag® system. More details and performance characteristics of these procedures can be found in publically available FDA resources^28^.

### Trioplex real-time RT-PCR assay

The CDC Trioplex real-time RT-PCR assay received an EUA from the US FDA in March 2016 for the diagnosis of dengue, chikungunya, and Zika viruses in serum, CSF, and whole blood-EDTA and for the diagnosis of Zika virus in urine and amniotic fluid. This study reports the optimization and validation of testing detection of dengue, chikungunya, and Zika virus RNA in serum, urine, and whole blood-EDTA. The Trioplex real-time RT-PCR assay includes a set of published and unpublished oligonucleotide primers and dual-labeled hydrolysis Taqman® probes for in vitro qualitative detection of dengue (unpublished), chikungunya (unpublished), and Zika viruses^18^. All primers and probes are described in patent application No. PCT/US2017/023021. All oligonucleotides used in this assay were initially evaluated in silico using publicly available genomes^28^ and to complement this evaluation, 33 additional ZIKV complete genomes representing the African and Asian genotypes were screened. Two or less mismatches differentiating the African from the Asian genotypes were detected; however, the effect of these mismatches in strain detection has not been evaluated due to lack of contemporary clinical samples linked to transmission of the African genotype. The Trioplex assay follows conventional real-time RT-PCR where complimentary DNA (cDNA) is reverse transcribed from viral RNA present in the sample and amplified by PCR. The fluorophore-labeled hydrolysis probes bind to the amplified DNA target fragment and the intensity of the fluorescent signal is captured by a real-time PCR instrument: (ABI 7500 fast Dx (ThermoFisher) or QuantStudio Dx (ThermoFisher)). Target amplification is interpreted from the exponential increase in fluorescence per amplification cycle in contract to background signal. Two one-step real-time RT-PCR master mixes were validated in this study: SuperScript® III Platinum® One-Step qRT-PCR System without ROX (ThermoFisher) and qScript™ One-Step qRT-PCR kit, Low Rox™. Each virus-specific probe was labeled with a distinct fluorophore dye in order to allow the assay to be run in multiplex and singleplex formats. The dengue virus-specific primer set is designed to target the 5′-untrascribed region of all contemporary genotypes of dengue viruses without serotype discrimination, and the probe is labeled with 6-carboxyfluorescein (FAM) on the 5′ end and is quenched by BHQ-1 on the 3′ end. The chikungunya virus-specific primer set was designed to target the non-structural protein 1 gene from the Asian and East, Central, and South African (ECSA) genotypes^42^, and the probe is labeled with 6-carboxy-2′,4,4′,5′,7,7′-hexachlorofluorescein (HEX) on the 5′ end and is quenched by BHQ-1 on the 3′ end. The Zika virus-specific primer set was designed to target the envelope gene of the Asian genotype^18^, and the probe is labeled with Cal fluor red 610 (Texas Red) on the 5′ end and quenched by BHQ-2 on the 3′ end. The multiplex real-time RT-PCR reaction is assembled by mixing 10 µL of sample RNA with 12.5 µL of PCR master mix reaction buffer (SuperScript III or qScript), virus-specific primers to a final concentration of 1 µM, dengue-specific probe to a final concentration of 0.3 µM, chikungunya-specific probe to a final concentration of 0.15 µM, Zika virus-specific probe to a final concentration of 0.15 µM, and nuclease-free water to a final reaction volume of 25 µL in a 96-well optical PCR plate. Similarly, the singleplex real-time RT-PCR reaction is assembled with the same components mentioned above at the same concentrations but compensating with nuclease-free water to reach the final reaction volume of 25 µL. Standard, non-fast, cycling protocols were selected and fluorescence capture is set to detect light emissions through the FAM, VIC (channel analogous to HEX), and Texas Red (channel analogous to Cal fluor red 610) in each well if running the assay in multiplex format or a single channel per well if running the assay in a singleplex format. Thermocylcling protocols were as follows: reverse transcription (RT) at 50 °C for 30 min, RT inactivation at 95 °C for 2 min (SuperScript III) or 5 min (qScript), fluorescence detection at 95 °C for 15 s, and annealing at 60 °C for 1 min. This assay also includes an internal control reaction that targets human endogenous ribonuclease P (RP), which is used to ensure the extracted test specimen contains amplifiable RNA. The RP reaction is run separately in singleplex format with the same sample RNA and with oligonucleotides that have been published previously^29^. Amplification curves were evaluated for each target virus individually and the threshold line was placed above overt background signal in the initial phase of the exponential phase of the curve. The threshold line position is placed individually for each target virus and the point (cycle) in which the amplification curve intersects the threshold line is referred to as the CT value. Although amplification curves with CT values > 38 may represent true virus-specific RNA detection, these high values are less than 95% reproducible or may represent erratical amplification curves and are therefore, considered negative. Trioplex assay data are interpreted as follows: a sample with target virus CT < 38 and RP reaction CT < 38 is considered positive, a sample with target virus CT ≥ 38 and RP reaction CT < 38 is considered negative, a sample with target virus CT < 38 and RP reaction CT ≥ 38 is considered positive, a sample with target virus CT ≥ 38 and RP reaction CT ≥ 38 is considered invalid. Every Trioplex assay run must include the following controls: Human Specimen Control (HSC) (serves as an RNA extraction control, as well as it serves as the positive control for the RP internal control reaction and as the non-template control (NTC) for the Trioplex assay reaction), PCR-grade water that serves as the overall negative control, and target-specific RNA (DENV-1-4 mix, CHIKV, and ZIKV) that serves as PCR positive control for each target virus. All evaluation data presented in this study are compliant with this assay format and all positive and negative controls were included. More details and performance characteristics of these procedures can be found in publically available FDA resounces^28^.

### Limit of detection

In order to determine the LoD of the assay in every standardized modality, live infectious virus was suspended in normal human serum, urine, or whole blood-EDTA to prepare dilution panels. High titer stocks (>10^6^ pfu/ml) of DENV-1 Puerto Rico strain 1998, DENV-2 Puerto Rico strain 1998, DENV-3 Puerto Rico strain 2004, DENV-4 Puerto Rico strain 1998, CHIKV Puerto Rico strain 2014, and ZIKV French Polynesia strain 2013 amplified in tissue culture were used. Commercial normal human serum (Corning), fresh urine pooled from healthy pre-tested individuals, or frozen whole blood-EDTA from healthy pre-tested individuals obtained from the Blood Systems Research Institute were spiked with virus and 8–12 serial dilutions (1:10) were performed as an initial range finding study. Viral RNA was extracted from each dilution in triplicate and the Trioplex assay was performed to determine the tentative LoD; the last dilution were all three replicates amplify at a CT < 38. The tentative LoD dilution, as well as the 10-fold dilution above the LoD and the 10-fold dilution below the LoD were selected for confirmation of the true LoD. Viral RNA was subsequently extracted from 20 replicates per dilution and tested with the Trioplex assay. The confirmed LoD is determined to be the last dilution where 95% of the replicates are positive (CT < 38). Serial dilutions prepared with heat-inactivated virus were tested but did not generate representative amplification curves and reproducible results. The corresponding risk assessments for biological containment and biosafety were performed in each laboratory prior to testing. Dengue and Zika virus dilutions and RNA extractions were processed and in BSL-2 laboratories, whereas chikungunya virus dilutions and RNA extractions were processed in BSL-3 laboratories.

### Specificity studies

The specificity of the Trioplex real-time RT-PCR assay was evaluated by testing the detection of target and non-target viral RNA. Specificity of target virus detection was evaluated by suspending quantified stocks of DENV-2, CHIKV, and ZIKV in a pool of human serum collected from patients with febrile illness that tested negative for all target viruses. Multiple combinations of a single virus or a mix of multiple viruses, e.g., ZIKV, ZIKV + CHIKV, DENV + CHIKV + ZIKV, were suspended at concentrations ranging from 10^5^ to 10^8^ GCE/mL. Five replicates of single virus suspensions, 10 replicates of mixed virus suspensions, and 50 mock-suspended replicates were tested. Cross-reactivity with non-target virus was tested by suspending laboratory stocks of West Nile virus NY99 strain, Yellow Fever virus 17D strain, and St. Louis encephalitis virus MSI-7 strain in normal human serum. Virus strains were obtained from the CDC Dengue Branch archival reference collection. RNA was extracted using the MagNA Pure LC 2.0 small volume protocol and tested in duplicates at three 10-fold dilutions near the LoD with the Trioplex assay in multiplex format.

### Clinical validation

The performance of the Trioplex real-time RT-PCR assay was evaluated with clinical diagnostic specimens received at the CDC Dengue Branch through the SEDSS. Positive and negative percent agreement with previous diagnostic determination was achieved by testing 155 randomly selected serum specimens including 20 ZIKV positive cases, 82 DENV positive cases (24 DENV-1, 22 DENV-2, 25 DENV-3, and 11 DENV-4), 13 CHIKV positive cases, and 40 negative cases from symptomatic patients. All specimens were retrieved from the CDC Dengue Branch positive specimen archival collection stored at −70°. Previous diagnostic determination was achieved by testing DENV cases with the CDC DENV-1-4 real-time RT-PCR assay^29^, CHIKV cases with a CHIKV nSP1 singleplex real-time RT-PCR LDT^42^, and ZIKV cases with ZIKV NS3 singleplex real-time RT-PCR LDT^18^. Clinical performance across specimen types was also evaluated. A stratified random sampling method was utilized to select the specimens for this study. The SEDSS database was queried to obtain all patients tested during the months of July, August, and September 2016 at the CDC Dengue Branch with paired serum, urine, and whole blood-EDTA collected concurrently in the acute stage (days 0–7 post onset of symptoms) and in which Zika virus RNA was detected in any of the specimens. Also, a paired convalescent serum sample was required each patient and tested positive for anti-Zika IgM using the CDC Zika IgM MAC ELIZA^14^. A total of 373 patients were identified and 1091 samples were retrieved from the frozen archive collection and re-tested for this study. All specimens were thawed to room temperature, viral RNA was extracted using the MagNA Pure 96 external lysis, small volume protocol, and tested with the Trioplex real-time RT-PCR assay in multiplex format. ZIKV RNA was quantified using an RNA transcript standard curve.

### Data availability

The Trioplex real-time RT-PCR assay includes a set of published and unpublished oligonucleotide primers and dual-labeled hydrolysis Taqman® probes for in vitro qualitative detection of dengue (unpublished), chikungunya (unpublished), and Zika viruses^18^. All primers and probes are described in patent application No. PCT/US2017/023021 and are available from the corresponding author upon request. Other relevant data supporting the findings of the study are available in this article and its Supplementary Information files, or from the corresponding author upon request.

## Electronic supplementary material

Supplementary Information(PDF 153 kb)
