# Supplementary material for: Performance of the Trioplex real-time RT-PCR assay for detection of Zika, dengue, and chikungunya viruses
Source: Nat Commun. 2018 Apr 11;9:1391. doi: 10.1038/s41467-018-03772-1 (PMC5895813; doi:10.1038/s41467-018-03772-1)
Supplement: Supplementary file 1 — Supplementary Information(PDF 153 kb) [file 41467_2018_3772_MOESM1_ESM.pdf]

SUPPLEMENTARY INFORMATION

Supplementary Table 1. ZIKV RNA stability in whole blood

	CT Values						
**Sample #**	**Fresh**	**3 days (4˚C)**	**9 days (4˚C)**	**10 days (-20˚C)**	**Average**	**Std. Dev.**	**Variance**
1	30.05	29.60	28.58	29.68	29.48	0.63	0.30
2	34.92	32.66	32.02	32.19	32.95	1.34	1.35
3	26.31	25.24	25.59	27.13	26.07	0.84	0.53
4	25.50	25.17	25.15	26.78	25.65	0.77	0.44
5	36.22	33.61	34.08	32.96	34.22	1.41	1.49
6	28.33	23.88	24.08	24.36	25.16	2.12	3.37
7	29.20	30.79	29.05	28.66	29.43	0.94	0.66
8	32.15	32.11	30.92	31.11	31.57	0.65	0.32
9	26.33	27.18	27.78	27.35	27.16	0.61	0.28
10	34.96	35.49	34.25	33.91	34.65	0.71	0.38
11	33.61	33.00	28.23	31.93	31.69	2.41	4.36
12	26.85	26.98	32.50	26.30	28.16	2.91	6.35
13	32.79	32.68	33.36	35.61	33.61	1.37	1.40
14	34.31	34.98	33.22	34.65	34.29	0.76	0.44
15	32.82	35.29	25.18	32.60	31.47	4.37	14.31
16	24.33	24.32	33.35	25.42	26.86	4.36	14.26
17	28.76	28.62	30.42	33.36	30.29	2.20	3.64
18	31.34	30.97	33.06	32.67	32.01	1.01	0.77
19	34.07	33.51	33.61	35.89	34.27	1.11	0.92
20	35.41	36.33	30.69	35.82	34.56	2.61	5.10
21	27.88	28.25	30.22	26.84	28.30	1.41	1.50
22	30.75	31.87	34.36	31.26	32.06	1.60	1.92
23	35.53	35.93	31.94	33.60	34.25	1.85	2.55
24	32.80	33.75	30.68	31.86	32.27	1.31	1.29
25	31.12	31.65	29.09	30.30	30.54	1.11	0.93
26	30.06	31.81	29.13	28.61	29.90	1.41	1.48
27	28.30	27.33	33.44	27.24	29.08	2.95	6.52
28	34.11	31.42	33.00	33.91	33.11	1.23	1.13
29	33.14	34.25	33.40	34.74	33.88	0.74	0.41
30	35.35	35.26	31.09	33.33	33.76	2.01	3.02
31	30.74	35.22	33.50	30.01	32.37	2.42	4.41
32	32.02	35.81	32.87	31.29	33.00	1.98	2.95
33	32.91	35.43	31.77	31.78	32.97	1.72	2.23
34	34.05	33.68	negative	32.41	33.38	0.86	0.49

Supplementary Table 2. Trioplex Assay cross-reactivity with non-target arboviruses

Virus	Strain	ZIKV result	DENV result	CHIKV result
West Nile virus	NY99	No cross-reactivity	No cross-reactivity	No cross-reactivity
yellow fever virus	17D	No cross-reactivity	No cross-reactivity	No cross-reactivity
St. Louis encephalitis virus	MSI-7	No cross-reactivity	No cross-reactivity	No cross-reactivity
Zika virus*	French Polynesia 2013	n/a	No cross-reactivity	No cross-reactivity
dengue virus*	Representatives from all 4 serotypes	No cross-reactivity	n/a	No cross-reactivity
chikungunya virus*	Puerto Rico	No cross-reactivity	No cross-reactivity	n/a

***** Cross-reactivity findings for these three viruses were extrapolated from data presented in limit of detection, contrived specimen and archived clinical specimen evaluations. N/A not applicable.

Supplementary Table 3. Zika virus detection in clinical specimens

	**Serum**	**Urine**	**Whole blood-EDTA**
**CT**	31.96 median<br>3.22 std. dev.<br>(31.10, 31.82) 95% CI	32.58 median<br>3.98 std. dev.<br>(31.87, 32.76) 95% CI	31.41 median<br>3.82 std. dev.<br>(30.59, 31.47) 95% CI
**GCE/mL**	4.72E+04 median<br>1.03E+01 std. dev.<br>(5.22E+04, 8.79E+04) 95% CI	2.94E+04 median<br>1.31E+01 std. dev.<br>(2.54E+04, 4.50E+04) 95% CI	7.08+04 median<br>1.52E+01 std. dev.<br>(6.98E+04, 1.31E+05) 95% CI
	**Serum RP**	**Urine RP**	**Whole blood-EDTA RP**
**CT**	25.50 median<br>23.35-37.26 range<br>(28.11, 28.69) 95% CI	35.54 median<br>21.85-37.59 range<br>(31.90, 32.71 95% CI	24.27 median<br>20.36-30.98 range<br>(24.16, 24.60) 95% CI
